# Identifying Behavioral Phenotypes of Loneliness and Social Isolation with Passive Sensing: Statistical Analysis, Data Mining and Machine Learning of Smartphone and Fitbit Data

**DOI:** 10.2196/13209

**Published:** 2019-07-24

**Authors:** Afsaneh Doryab, Daniella K Villalba, Prerna Chikersal, Janine M Dutcher, Michael Tumminia, Xinwen Liu, Sheldon Cohen, Kasey Creswell, Jennifer Mankoff, John D Creswell, Anind K Dey

**Affiliations:** 1 School of Computer Science Carnegie Mellon University Pittsburgh, PA United States; 2 School of Engineering and Applied Sciences The University of Virginia Charlottesville, VA United States; 3 Department of Psychology Carnegie Mellon University Pittsburgh, PA United States; 4 School of Education University of Pittsburgh Pittsburgh, PA United States; 5 Paul G Allen School of Computer Science and Engineering University of Washington Seattle, WA United States; 6 Information School University of Washington Seattle, WA United States

**Keywords:** mobile health, loneliness, machine learning, statistical data analysis, data mining, digital phenotyping

## Abstract

**Background:**

Feelings of loneliness are associated with poor physical and mental health. Detection of loneliness through passive sensing on personal devices can lead to the development of interventions aimed at decreasing rates of loneliness.

**Objective:**

The aim of this study was to explore the potential of using passive sensing to infer levels of loneliness and to identify the corresponding behavioral patterns.

**Methods:**

Data were collected from smartphones and Fitbits (Flex 2) of 160 college students over a semester. The participants completed the University of California, Los Angeles (UCLA) loneliness questionnaire at the beginning and end of the semester. For a classification purpose, the scores were categorized into high (questionnaire score>40) and low (≤40) levels of loneliness. Daily features were extracted from both devices to capture activity and mobility, communication and phone usage, and sleep behaviors. The features were then averaged to generate semester-level features. We used 3 analytic methods: (1) statistical analysis to provide an overview of loneliness in college students, (2) data mining using the Apriori algorithm to extract behavior patterns associated with loneliness, and (3) machine learning classification to infer the level of loneliness and the change in levels of loneliness using an ensemble of gradient boosting and logistic regression algorithms with feature selection in a leave-one-student-out cross-validation manner.

**Results:**

The average loneliness score from the presurveys and postsurveys was above 43 (presurvey SD 9.4 and postsurvey SD 10.4), and the majority of participants fell into the high loneliness category (scores above 40) with 63.8% (102/160) in the presurvey and 58.8% (94/160) in the postsurvey. Scores greater than 1 standard deviation above the mean were observed in 12.5% (20/160) of the participants in both pre- and postsurvey scores. The majority of scores, however, fell between 1 standard deviation below and above the mean (pre=66.9% [107/160] and post=73.1% [117/160]).

Our machine learning pipeline achieved an accuracy of 80.2% in detecting the binary level of loneliness and an 88.4% accuracy in detecting change in the loneliness level. The mining of associations between classifier-selected behavioral features and loneliness indicated that compared with students with low loneliness, students with high levels of loneliness were spending less time outside of campus during evening hours on weekends and spending less time in places for social events in the evening on weekdays (support=17% and confidence=92%). The analysis also indicated that more activity and less sedentary behavior, especially in the evening, was associated with a decrease in levels of loneliness from the beginning of the semester to the end of it (support=31% and confidence=92%).

**Conclusions:**

Passive sensing has the potential for detecting loneliness in college students and identifying the associated behavioral patterns. These findings highlight intervention opportunities through mobile technology to reduce the impact of loneliness on individuals’ health and well-being.

## Introduction

### Background

Loneliness in the United States and across the world is rising to an epidemic level [1]. According to the latest US Loneliness Index Report [2], nearly half of Americans report high levels of loneliness with an average loneliness score of 43.9. Of those surveyed, 46% reported sometimes or always feeling lonely and 47% reported feeling separated from others. The highest levels of loneliness were found among young adults aged 18 to 22 years who had an average loneliness score of 48.3. Loneliness is defined as a negative emotional experience caused by a discrepancy between the desired and achieved social contact [3] or perceived social isolation [1,4]. As opposed to aloneness, which is a state of being physically alone, loneliness relates to a subjective feeling and can occur in individuals despite having social relationships or being around others [5,6].

Social relationships are intricately tied to individuals’ health, and a lack of social connection has an adverse impact on health and well-being [7,8]. In a landmark systematic review and meta-analysis of 148 studies examining social relationships and mortality risk, Holt-Lunstad et al [9] found that older adults with stronger social relationships had a 50% increased likelihood of survival than those with weaker social relationships. Subsequent research by this group found that social isolation, loneliness, and living alone were greater risks for mortality than obesity [10]. Importantly, loneliness has also been associated with higher risk for developing depression and other mental health problems [10].

Given the significance of loneliness on health and well-being outcomes, the goal of this study was to detect and understand loneliness through behavioral signals collected from smartphone and wearable devices. Wide usage of mobile devices provides an opportunity to passively collect daily behavioral traces that relate to mental health and well-being over a long period of time. We were interested in understanding (1) how well we could detect if someone was lonely by analyzing their daily digital behavioral signals and (2) what behavioral patterns were associated with loneliness.

### Related Work

Pulekar et al [11] studied the first question in a small study with 9 college students over 2 weeks. Data logs of social interactions, communication, and smartphone activity were analyzed to detect loneliness and its relationship with personality traits. The study reports 90% accuracy in classifying loneliness using the smartphone features that were mostly correlated with the loneliness score. However, the small sample size, the short duration of the data collection phase, and missing details in the machine learning approach, especially the classification evaluation, make the results difficult to generalize and build on. Sanchez et al [12] used machine learning to infer the level of loneliness in 12 older adults who used a mobile app for one week. Call logs and global positioning system (GPS) coordinates were collected from the phones. A total of 4 models for family loneliness, spousal loneliness, social loneliness, and existential crisis were built with a reported accuracy of 91.6%, 83.3%, 66.6%, and 83.3%, respectively. However, similar to the results of the study by Pulekar et al, these results may fail to generalize because of the small sample and short duration of data collection.

A few studies have explored the second question using correlation analysis to understand relationships between single behavioral signals, such as level of physical activity, mobility, social interactions, and loneliness [13-15]. Wang et al [14] analyzed smartphone data collected from 40 students over a spring semester and found negative correlations between loneliness and activity duration for day and evening times, traveled distance, and indoor mobility during the day. A related study from the same group found statistically significant correlations (*P*<.01) between kinesthetic activities and change in loneliness but no relationship between loneliness and sleep duration, geospatial activity, or speech duration [13]. Gao et al [15] found that people with higher levels of loneliness made or received fewer phone calls and used certain types of apps, such as health and fitness, social media, and Web browsing, more frequently than those with low levels of loneliness. Our data mining approach, in addition to providing similar behavioral features to those reported by Wang et al [14], presents an innovative method for extracting the combined behavioral patterns in our participant population. For example, we can observe that compared with students with a low level of loneliness, students with a high level of loneliness unlock their phones in different time segments during weekends, spend less time off-campus during evening hours on weekends, and socialize less during evening hours on weekdays. To our knowledge, this study introduces, for the first time, an approach toward extracting combined behavioral patterns through data mining and their associations with a mental health outcome, such as loneliness, from passive sensing data.

## Methods

### Recruitment and Data Collection

Data collection was done as part of a campus-wide study at an American research university in the state of Pennsylvania to assess students’ health and well-being. The participants were first-year undergraduate students recruited via advertisement on student mailing lists and Facebook groups. An identity document (ID) was assigned to each participant and documents connecting the ID and participant’s name and demographics were kept separate. The data on the phone were anonymous and only identifiable through the participant’s device ID. All data collection procedures in this study were approved by the university’s Institutional Review Board (IRB: STUDY2016_00000421), including the collection of location data. Students were invited to an initial appointment in our lab to be screened for eligibility, provide written informed consent to participate in the study, and allow us to collect their data. At this appointment, participants downloaded the open-source AWARE data collection app [16] that was developed in our lab to track sensor data from their own Android or iOS smartphones and they received a Fitbit Flex 2 to track steps and sleep. Later, the students completed Web-based questionnaires for an initial assessment of their health and well-being. At the end of the study, students filled out the same questionnaires for post measurements. Out of the 188 first-year college students initially enrolled, 160 (61% female, 57% Asian, 34% white, 9% Hispanic, and 5% black) completed all pre- and postsemester surveys. Participants were informed about the purpose of the study during the initial appointment session. There was no deception or omission of study aims to the participants.

Data were collected passively from their smartphone and Fitbit devices and were continuously recorded over 16 weeks of the study (1 semester that was the participants’ second semester at the university). The AWARE framework [17] is an open-source data collection app with supporting backend and network infrastructure, which collects sensor data unobtrusively from students’ smartphones. It supports both Android and iOS platforms and can be downloaded from the App and Play stores. AWARE enabled us to record nearby Bluetooth addresses, Wi-Fi, location, phone usage (ie, when the screen status changed to on or off and locked or unlocked), and call and short message service (SMS) text messaging logs. The participants were asked to keep their Bluetooth and Wi-Fi on during the study. To assess calls to close contacts, we asked the participants to provide phone numbers of family members, friends on campus, and friends off campus that they most frequently contact. We also used a conversation plugin for AWARE (same as the one used by Wang et al [14]), which makes audio inferences, such as silence, voice, noise, or unknown. Furthermore, we equipped the participants with a Fitbit Flex 2 wearable activity tracker that records the number of steps taken and sleep status (asleep, awake, restless, or unknown). Students were instructed to wear the device on their nondominant hand. We chose Flex 2 based on a combination of factors including simplicity, waterproofness, battery life, and price. Fitbit Flex has shown to have moderate validity to track activities compared with ActiGraph [16]. Calls and phone usage were event-based sensor streams, whereas Bluetooth, Wi-Fi, location, sleep, and steps were sampled as time series. These time series data streams were sampled at different rates because of the capabilities of the hardware being used. Bluetooth and location coordinates were collected at 1 sample per 10 min, sleep at 1 sample per min, and steps at 1 sample per 5 min. Data from AWARE were deidentified and automatically transferred over Wi-Fi to our backend server on a regular basis, and data from the wearable Fitbit were retrieved using the Fitbit app programming interface (API) at the end of the study. The participants were asked to keep their phone and Fitbit charged and with them at all times.

### Survey Data Processing

To assess loneliness, we used the revised University of California, Los Angeles (UCLA) loneliness scale, a well-validated and commonly used measure of general feelings of loneliness [18]. The participants provided ratings for each of the 20 questions (Textbox 1) using a scale of 1 (never) to 4 (always). A total of 9 items were reverse scored before all items were summed to create a total score. The total loneliness scores ranged from 20 to 80 with higher scores indicating higher levels of loneliness. As there is no standard cutoff for loneliness scores in the literature, each study has created arbitrary categorizations including the categories proposed in the study by Cacioppo et al [1]: High loneliness is defined as scoring 44 or higher, low loneliness is defined as scoring less than 28, and scores between 33 and 39 represent the middle of the spectrum. Although we could adapt these categories, our goal was to do a binary classification to detect the level of loneliness, which required dividing the loneliness scores into 2 categories. We also wanted to create cutoff scores that were independent of the population distribution but represented conceptual indicators of loneliness. Thus, as the answer choices provided were 1=never, 2=rarely, 3=sometimes, and 4=often, we determined that scores of 40 and below indicated that the participants were rarely or never experiencing loneliness and scores of 41 and above would indicate at least sometimes experiencing loneliness (a participant that answered rarely (score=2) to all 20 questions would have a total score of 40, suggesting that 40 indicates that the participant is rarely experiencing loneliness). We, therefore, used 40 as the cutoff point where the scores of 40 and below were categorized as no to low loneliness and the scores above 40 were categorized as moderate to high loneliness. For simplification, we refer to the no to low loneliness category as low loneliness and the moderate-to-high loneliness category as high loneliness. These categories were used as ground truth labels in our machine learning pipeline to infer the loneliness level. Although this choice can be replicated in other similar studies, further sensitivity analyses should be done to determine the optimal cutoff point for the UCLA scale.

**List of questions used in the University of California, Los Angeles, loneliness scale (questions marked with R were reverse scored).**

R1. How often do you feel that you are in tune with the people around you?

2. How often do you feel that you lack companionship?

3. How often do you feel that there is no one you can turn to?

4. How often do you feel alone?

R5. How often do you feel part of a group of friends?

R6. How often do you feel that you have a lot in common with the people around you?

7. How often do you feel that you are no longer close to anyone?

8. How often do you feel that your interests and ideas are not shared by those around you?

R9. How often do you feel outgoing and friendly?

R10. How often do you feel close to people?

11. How often do you feel left out?

12. How often do you feel that your relationships with others are not meaningful?

13. How often do you feel that no one really knows you well?

14. How often do you feel isolated from others?

R15. How often do you feel you can find companionship when you want it?

R16. How often do you feel that there are people who really understand you?

17. How often do you feel shy?

18. How often do you feel that people are around you but not with you?

R19. How often do you feel that there are people you can talk to?

R20. How often do you feel that there are people you can turn to?

### Loneliness in College Students: Statistical Analysis

As the first step, we analyzed the distribution of loneliness among our participants. As mentioned, we categorized the UCLA loneliness scores into low (≤40) and high (>40) levels of loneliness. We then calculated the distribution of the overall scores as well as the distribution of responses to each question in the UCLA loneliness scale. This analysis helps identify the common response level to each question. Furthermore, we calculated the differences between the pre- and postsemester loneliness scores to understand the change in loneliness across the semester. We repeated this analysis with each question and measured the amount of change in students’ responses. We showed the distribution of questions being rated the same, above, or below the presemester loneliness in the post measurements, thus identifying the items that were more likely to change than others over time.

### Behavior Patterns of Loneliness: Data Mining Analysis

In addition to capturing the relations between each behavioral feature and loneliness, we were also interested in extracting combined behavioral patterns that were associated with loneliness. We measured the proportion of our study population that was covered by these combinations of behavioral patterns and discussed the technological implications of these observations. We also explored associations between responses to individual questions and level of loneliness as well as behavioral features and level of loneliness.

We applied Apriori [19], a well-known frequent itemset algorithm for discovering associations among items in transactional datasets, to extract patterns between the overall loneliness level and combined questions as well as combined behavioral patterns that were most associated with the level of loneliness. Apriori extracts patterns in 2 steps: it first generates a set of frequent items that appear together and then extracts association rules that explain the relationship between those frequent items. The extracted rules must satisfy a degree of support and confidence in the dataset. For example, let A and B be 2 sets of items. An association (A→B) exists if items in A and B frequently appear together in transactions. Support is the percentage of transactions that contain both A and B, whereas confidence is the percentage of transactions containing A that also contain B [20], that is, support (A→B)=P(AUB) and confidence (A→B)=P(B|A). Note that the notation P(AUB) indicates the probability that a transaction contains the union sets of A and B (ie, it contains every item in A and B). This should not be confused with P(A or B), which indicates the probability that a transaction contains either A or B [20].

To simplify the pattern mining process, we further discretized the behavioral features into categories of low, moderate, and high using binning with equal frequency. We then applied Apriori on the selected feature set generated in the machine learning process described in the following section.

### Detection of Loneliness Level and Change in Loneliness: Machine Learning Analysis

**Figure 1:**
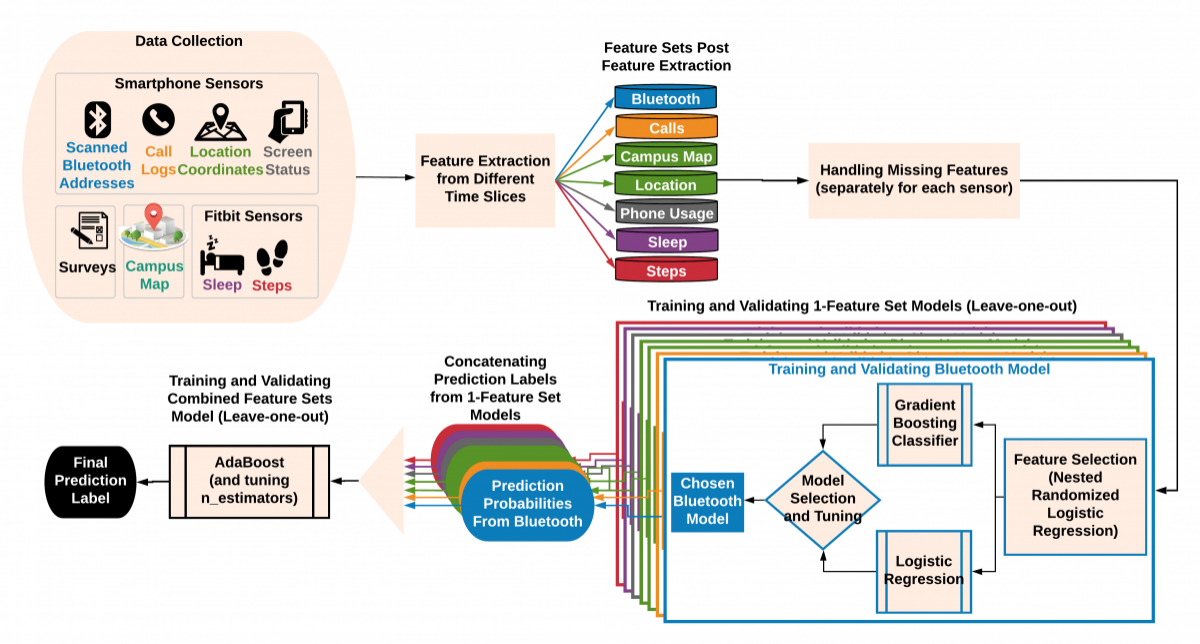
Machine learning pipeline including data collection, feature extraction, training and validation, and final output.

To explore the use of passive sensing in inferring the state of loneliness, we defined loneliness detection as a binary classification problem, where the aggregated behavioral data over the semester were used as feature vectors to infer the level of loneliness (low or high). We followed the same categorization described earlier to label loneliness scores as low or high. Our modeling pipeline (Figure 1) handles each sensor separately (called 1-feature set) during the training and validation and provides a combined final classification outcome at the end. Using the 1-feature sets provides the possibility to examine the predictive power of each sensor alone and combined. Specifically, our approach comprised the following processes:

1. Passive data processing and feature extraction
2. Handling missing values
3. Training and validating models that use only 1-feature set for each of the following 7 feature sets: Bluetooth, calls, campus map, location, phone usage, sleep, and steps
4. Obtaining the final label for the outcome by combining detection probabilities from 1-feature set models

The processes are described in the following sections.

### Passive Data Processing and Feature Extraction

**Figure 2:**
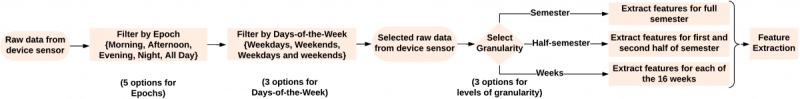
Raw data from each sensor was preprocessed and then filtered by an epoch and a days-of-the-week option.

Our data included time series data from Bluetooth, calls, SMS, Wi-Fi, location, phone usage, steps, and sleep. These sensing channels have the potential to capture daily behavioral patterns related to loneliness, namely, mobility and activity patterns, communication and social interaction, and sleep. We developed a generic and flexible feature extraction component (FEC) [21] to extract features from raw sensor data collected from the smartphones and Fitbit devices. FEC computes features from timestamped streams of data in specified time segments ranging from 5 min to several months. From the data streams, FEC extracts a set of common statistical features, such as minimum, median, mean, maximum, and standard deviation, as well as more complex behavioral features, such as movement regularity and travel distance. Each feature from every time series data was extracted from 45 time segments illustrated in Figure 2. First, we fetched all the available data (spanning over multiple days of the study) from a certain epoch or time of the day (all day; night, ie, 12 am-6 am; morning, ie, 6 am-12 pm; afternoon, ie, 12 pm-6 pm; and evening, ie, 6 pm-12 am) and for certain days of the week (all days of the week; weekdays only, ie, Monday-Friday; weekends only, ie, Saturday-Sunday). Then, we calculated features from these data aggregated over different levels of granularity (eg, whole semester, two-halves of the semester, and weekly). As there are 5 epochs, 3 days-of-the-week segmentations, and 3 levels of granularity, we get 5×3×3=45 time segments. Note that the 2 halves of the semester are not perfect halves. For simplicity, we refer to week 1 to week 6 (before midterms) as the first half and week 7 to week 16 (midterms and after midterms) as the second half. In total, we extracted 77,805 features from our time series data in combination with different time segments. The source code for extracting these features will be available upon request. The following describes features extracted in each behavior category.

### Mobility and Physical Activity Features

Features related to mobility were extracted from the GPS coordinates including location variance (sum of the variance in latitude and longitude coordinates), log of location variance, total distance traveled, average speed, and variance in speed. We followed the approach in the study by Tan et al [22], which used the Lomb-Scargle method [23] to extract movement regularity from location patterns that follow a 24-hour cycle. Additional features were extracted through the following process:

1. We calculated the movement speed from the distance covered and time elapsed between 2 samples. Samples with speed >1 km/h were labeled as moving, else static.
2. Samples labeled as static were clustered using density-based spatial clustering of applications with noise (DBSCAN), a density-based clustering algorithm [24] to find frequent places visited by the participant and labeled as global or local clusters. DBSCAN efficiently groups nearby spatial points together and distinguishes outlier points. Unlike other clustering algorithms, such as k-means, DBSCAN does not require knowing the number of clusters a priori. It is able to find inner clusters (clusters surrounded by other clusters) and is robust to outliers and noise. Global clusters were extracted using all data and local clusters were extracted when data were split into daily time segments described earlier.

These steps allowed us to extract the number of frequent places, number of transitions between places, radius of gyration [25], time spent at top 3 (most frequent) local and global clusters, percentage of time spent moving, and percentage of time spent in infrequent or rarely visited locations (labeled as −1 by DBSCAN). We also calculated statistics related to the length of stay at clusters, such as maximum, minimum, average, and standard deviation of the length of stay at local and global clusters, as well as location entropy and normalized location entropy across local and global clusters. Location entropy is higher when time is spent evenly across frequent places. Calculating features for both local and global clusters allowed us to capture different behaviors related to the user’s overall location patterns (global) and the user’s location patterns within a time slice (local). For example, time spent at top 3 global and local clusters capture the time spent at user’s overall frequent places and user’s frequent location in a particular time slice (eg, mornings on weekends). We assumed the place most visited by the participant at night to be their home location.

To approximate the home location, we performed abovementioned steps (1) and (2) on the location coordinates from all nights (12 am to 6 am) and assumed the center of the most frequented cluster to be the participant’s home location center. As we do not know the radius of the home, we calculated two home-related features: time spent at home assuming home to be within 10 meters of the home location center and time spent at home assuming home to be within 100 meters of the home location center. We also analyzed the user’s location patterns in relation to their college campus. First, we obtained a campus map of the participants’ university. Then, we marked out the campus boundary and different types of buildings on campus by creating polygons on Google Maps using an online Geographical Information System. We annotated 6 categories of buildings and spaces—2 different houses that hold the most social events, student apartments, residential halls, athletic facilities, and green spaces. As academic buildings in this university are often collocated with other spaces, we assumed any on-campus space not belonging to these 6 categories to be an academic building. For every location sample, we assigned 1 of 8 location category labels (6 building/space types, academic, off campus). Then, the following features were extracted for each type of space: time spent at each location type in min; percentage of time spent at each location type; number of transitions between different spaces; number of bouts (or continuous periods of time) at space; number of bouts during which a participant spends 10, 20, or 30 min at the same space; and minimum, maximum, average, and standard deviation of the length of bouts at each space. The campus map features also included 2 multimodal features—study duration and social duration. These features fused data from location, phone usage, audio, and steps sensors.

Study duration was calculated by fusing location type labels with data from the phone usage and steps sensors. A participant was assumed to be studying if they spent 30 min or more in an academic building while being sedentary (fewer than 10 steps) and having no interaction with their phone. Social duration was calculated by fusing location type labels with data from the audio sensor. A participant was assumed to be social if they spent 20 min or more in any of the residential buildings or green spaces and the audio sensor inferred human voice or noise for 80% or more of that time. Other activity- and mobility-related features were extracted from the step counts collected by Fitbit. We calculated the total number of steps and the maximum number of steps taken in any 5-min period. Other features were extracted from bouts, where a bout is a continuous period of time during which a certain characteristic is exhibited. Examples of such features included the total number of active or sedentary bouts [26], and the maximum, minimum, and average length of active or sedentary bouts. We also calculated minimum, maximum, and average number of steps over all active bouts. Directly using the results from the study of Cacioppo et al [26], we determined that a bout is sedentary if the user takes less than 10 steps during each 5-min interval within the bout. As soon as the user takes more than 10 steps in any 5-min interval, they switch to an active bout.

### Communication and Interaction Features

We used call and SMS logs to extract features including the number and duration of incoming, outgoing, and missed calls and messages to everyone, to family members, to friends off campus, and to friends on campus, number of correspondents overall, and number of correspondents who are family members, friends off campus, or friends on campus. We also extracted phone usage features that related to both communication and Web-based interaction. We used the logs of screen status (eg, on, off, lock, and unlock) over time. We extracted the number of unlocks per min, total time spent interacting with the phone, total time the screen was unlocked, the hour of the days the screen was first unlocked or first turned on, the hour of the days the screen was last unlocked, locked, and turned on, and the maximum, minimum, average, and standard deviation of the length of bouts (or continuous periods of time) during which the participant was interacting with the phone and when the screen was unlocked. A participant is said to be interacting with their phone between when the screen status is unlock and when the screen status is off or lock.

As Bluetooth connections can be a proxy of social interaction, we also extracted features from Bluetooth by first classifying scanned Bluetooth devices into 3 groups of self (the participant’s own devices), related (devices belonging to the participant’s partner, roommates, or classmates), and others (unrelated devices). To classify scanned Bluetooth addresses into the 3 groups of self, related, and others, we did the following:

1. We calculated number of days each unique Bluetooth address was scanned at least once, that is, number_of_daysbti.
2. We calculated the average frequency of each unique Bluetooth address, that is, average_frequencybti = total_countbti / number_of_daysbti.
3. We Z-normalized the number_of_daysbti and average_frequencybti to give equal weight to both while optimizing score in step 4.
4. For each Bluetooth address, we computed score = number_of_daysbti + average_frequencybti.
5. We used K-means clustering to cluster score from step 4 for all Bluetooth addresses using K=2 and K=3.
6. The model with K=2 was chosen if the sum of squared distances between clustered points and cluster centers was smaller than what we got with K=3. Otherwise, we chose model with K=3.
7. If the model with K=2 was chosen, the cluster with higher scores contained the participant’s own devices (self), whereas the other cluster contained other people’s devices (others). If the model with K=3 was chosen, the cluster with the highest scores contained the participant’s own devices (self), the cluster with the lowest scores contained other people’s devices (others), and the remaining cluster contained devices of the participant’s partners, roommates, or officemates (related). Once the Bluetooth addresses scanned were clustered into self and others or self, related, and others, we extracted features including the number of unique devices, number of scans of the most and the least frequent device, and sum, average, and standard deviation of the number of scans of all devices. Each round included all devices (ignores clusters), self and related cluster (combined), and others cluster.

#### Sleep Features

Sleep features were extracted from the sleep inferences (eg, asleep, restless, awake, and unknown) over time returned by the Fitbit API. We calculated the number of asleep samples, number of restless samples, number of awake samples, weak sleep efficiency (the sum of the number of asleep and restless samples divided by the sum of the number of asleep, restless, and awake samples), strong sleep efficiency (the sum of the number of asleep samples divided by the sum of the number of asleep, restless, and awake samples), count, sum, average, maximum, and minimum length of bouts during which the participant was asleep, restless, or awake, and the start and the end time of the longest and the shortest bouts during which the participant was asleep, restless, or awake.

#### Feature Matrix

**Figure 3:**
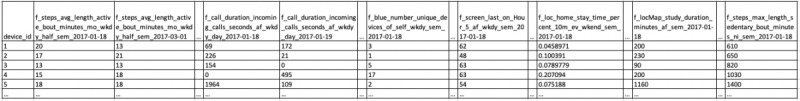
The schema of the feature matrix used in the machine learning pipeline (each column is a feature and each row is a sample per participant).

After feature extraction, we obtained a feature matrix for each of the 7 feature sets derived from different sensors. In each of these feature matrices, each sample or record contained features extracted from one student. We aggregated our features over different time segments (described in Figure 2): over different weeks, in the two-halves of the semester, and across the whole semester. The features from all these time segments were concatenated to form the feature vector for each student. A scheme of the feature matrix is shown in Figure 3. The coding schema is described in the Multimedia Appendix 1 and a sample of selected features is presented.

#### Handling Missing Values

We handled missing data on a 1-feature set basis: for each sensor, we removed a feature from the dataset if its value was missing for more than 30 participants, and we removed a participant from the dataset if 20% of their data were missing. The thresholds for removing data were determined empirically. We then imputed the remaining missing feature values with a −1. This was chosen because all feature values were above 0 and as such −1 could distinguish missing values. The same features calculated over different time segments were viewed independently, for example, if a feature was missing for a week for over 30 people, we removed that feature from that week only. As such, the number of samples that were used in training and validation for each feature set varied. For example, when training with semester-only features, the smallest feature set belonged to location (with 118 samples) and the largest sets were Bluetooth and phone usage (with 134 samples).

#### Building and Validating 1-Feature Set Models

Building and validating 1-feature set models followed 3 steps:

1. Feature selection
2. Training 2 algorithms, namely, logistic regression and gradient boosting, to build models of each feature set using selected features
3. Selecting the model with better accuracy

All these steps were done in a leave-one-student-out cross-validation, that is, at each step of training and feature selection, we built a separate model using data from n−1 students and tested it on the nth student. Note that the data for each student were represented as one sample in each feature set in the form of a vector.

#### Feature Selection

The wide range of behavioral features provides the possibility to draw insights into different types and granularity of behavior in relationship to loneliness. However, the large number of features makes it difficult for the classification algorithm to build a comprehensive model of data, especially when the size of the sample set (eg, number of participants) is proportionally smaller than the feature set. Therefore, we applied feature selection to reduce the number of features to a set that is representative of our data. We experimented with different feature selection methods including least absolute shrinkage and selection operator (LASSO) [27] and randomized logistic regression [28] that have been shown to perform well in selecting a stable set of features. In our case, because the number of features in each feature set was substantially larger than the sample size, those methods performed poorly, and the accuracy of models was low. We, therefore, applied randomized logistic regression in a hierarchical and nested manner on groups of features in each time segment. Randomized logistic regression creates several random subsamples of the training dataset, computes a logistic regression on each subsample, and selects features by optimizing their importance across all subsamples. We decomposed our feature space by grouping features from the same time segment and performed randomized logistic regression on each of these groups. The selected features from all groups (ie, all time segments) were then concatenated to give a new and much smaller set of features. Then, randomized logistic regression was performed again, this time on this new set of features to get the final selected features, thereby nesting the process. We call this method nested randomized logistic regression (NRLR). This method was performed in a leave-one-out manner such that the model used to detect an outcome for a person did not include those data from that person during the feature selection process.

Table 1 (columns 1-3) shows the number of features and number of samples for each feature set after handling missing values where all features were used as input to the training and validation. Table 1 (columns 4-7) shows a comparison between the features selected with LASSO and NRLR. Compared with LASSO, the average number of selected stable features (features selected in all cross-validation folds) is 3 times smaller in NRLR that substantially reduces the size of the feature vector.

**Table 1:** The list of feature sets with the number of features and data samples used in the machine learning pipeline after handling missing values and the number of selected features during the cross-validation process.

Feature set	Number of features	Number of samples	Number of features selected during cross-validation process			
			LASSO^a^		NRLR^b^	
			In all folds	In at least one fold	In all folds	In at least one fold
Bluetooth	3201	115	203	1026	278	1864
Calls	605	108	30	134	34	142
Campus map	16,381	111	66	455	12	161
Location	10,237	106	345	784	14	124
Screen	15,446	113	96	467	8	52
Sleep	5889	107	87	534	23	266
Steps	3055	107	270	485	0	8
Average	7831	110	157	555	53	374

^a^LASSO: least absolute shrinkage and selection operator.

^b^NRLR: nested randomized logistic regression.

#### Model Generation

For each feature set, we built a model of the selected features from that feature set to detect an outcome using 2 learning algorithms, namely, logistic regression and gradient boosting classifier. We chose logistic regression because it was used in our feature selection approach, and gradient boosting was chosen because it had shown to perform well on noisy datasets and learn complex nonlinear decision boundaries via boosting. Gradient boosting had been effectively used to detect similar outcomes in a previous study [29].

#### Model Selection

The generated models from logistic regression and gradient boosting were then evaluated by comparing their accuracy as a metric for postsemester and change in loneliness. The model that provided better accuracy was selected for the next step.

### Combining Detection Probabilities From 1-Feature Set Models to Obtain Combined Models

The chosen 1-feature set model in the previous step gave us detection probabilities for each outcome label. The detection probabilities from all 7 1-feature set models were concatenated into a single feature vector and given as input to an ensemble classifier, AdaBoost with gradient boosting as the base estimator, which then outputted the final label for the outcome. For the detection of postsemester loneliness, which is a binary classification, only the inferred probabilities of one of the classes (low or high) were concatenated, whereas for the detection of change in loneliness (multiclass classification: decreased loneliness, increased loneliness, and unchanged loneliness), the inferred probabilities of all classes were concatenated.

We also carried out a feature ablation study to analyze the effect that different feature sets had on the performance of the models, thereby understanding their salience. For this purpose, we concatenated detection probabilities from specific 1-feature set models instead of all 7 1-feature set models. We did this for all possible combinations of 1-feature set models to analyze the estimation power of each feature set in inferring loneliness level. There were 7 1-feature set models and 120 combinations of feature sets, as total combinations = combinations with 2 feature sets + ... + combinations with 7 feature sets = 120. We report the best accuracies obtained from these combinations.

### Measures

We summarize our measures used throughout the paper as follows:

- Preloneliness score—total UCLA loneliness score measured at the beginning of the semester
- Postloneliness score—total UCLA loneliness score measured at the end of the semester
- Increased score—when postloneliness score was greater than preloneliness score
- Decreased score—when postloneliness score was less than preloneliness score
- Unchanged score—when postloneliness score was equal to preloneliness score

Loneliness level (pre- or postsemester)—2 categories:

- Low loneliness (LL)—total UCLA scores of 40 and below
- High loneliness (HL)—total UCLA scores above 40

- Change in loneliness level from pre to post—3 categories:
- Decreased loneliness (DL)—loneliness level changed from high at presemester to low in postsemester
- Increased loneliness (IL)—loneliness level changed from low in presemester to high in postsemester
- Unchanged loneliness (UL)—loneliness level remained the same in presemester and postsemester

Machine learning measures:

- Baseline accuracy—percentage of samples belonging to the majority class (here HL). This percentage is compared with the classification output to measure the performance of the machine learning algorithms.
- Accuracy—percentage of correctly classified samples (1 per student)
- Precision—percentage of classified samples that actually belonged to a class, for example, HL or LL
- Recall—percentage of class samples that were accurately classified
- F1—harmonic mean of precision and recall
- MCC—a measure of quality of binary classification. The value is between −1 and 1 where 1 indicates a perfect prediction, 0 indicates no better than random prediction, and −1 indicates total disagreement between prediction and observation.

## Results

### Loneliness in College Students

**Figure 4:**
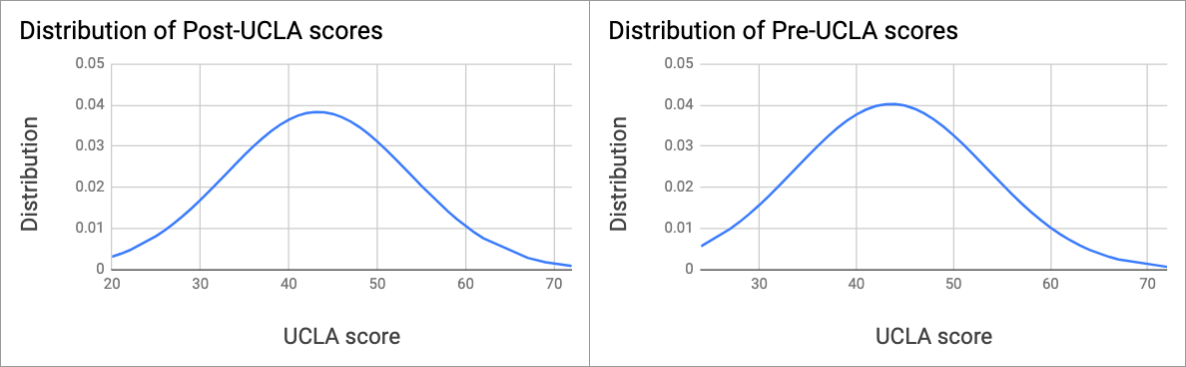
Distribution of presemester University of California, Los Angeles, scores (mean 43.6, SD 9.4) and postsemester University of California, Los Angeles, scores (mean 43.3, SD 10.4). UCLA: University of California, Los Angeles.

We analyzed the total UCLA loneliness scores for both presemester and postsemester surveys. The average score from the presemester surveys was above the cutoff point (mean 43.6, median 44, Q1=37, Q3=49, and SD 9.4) with 63.8% (102/160) of participants falling into the HL category (scores above 40). Similarly, at postsemester, the average loneliness score was above the cutoff (mean 43.3, median 43, Q1=37, Q3=50, and SD 10.4) with 58.8% (94/160) of participants falling into the HL category. Figure 4 shows the distribution of scores for both pre- and postsemester UCLA scores.

A paired test showed no significant difference between the two distributions (*P*=.73). We observed that the loneliness score for 12.5% (20/160) of participants was 1 standard deviation above the mean in both pre- and postsemester. The majority of scores, however, fell into the range between 1 standard deviation below and above the mean (pre=66.9% [107/160] and post=73.1% [117/160]). Table 2 shows the summary of the statistics.

**Table 2:** Statistics of high and low loneliness scores measured by University of California, Los Angeles, scale in pre- and postsurveys.

Category	Count		Average		Min		Max	
	Pre	Post	Pre	Post	Pre	Post	Pre	Post
All	160	160	43.6	43.3	—^a^	—	—	—
LL^b^	58 (36.2%)	66 (41.2%)	33.9	33.7	24	20	40	40
HL^c^	102 (63.8%)	94 (58.8%)	49.1	49.9	41	41	72	72

^a^Data not applicable.

^b^LL: low loneliness.

^c^HL: high loneliness.

The percentage of participants with a high postloneliness score was 5% lower than those with high preloneliness scores (58.75% vs 63.75%), indicating an overall lower loneliness rate among students at the end of the semester. Only 6 participants who had low loneliness scores in the presemester survey showed a high level of loneliness in the postsemester survey, whereas 7 participants had an HL score in the presemester survey but an LL score in the postsemester survey. The average increase and decrease were 6 and 7 points, respectively. Overall, 17.5% (28/160) of the participants reported a more than 6-point increase in their postloneliness scores, 18.8% (30/160) reported a more than 7-point decrease, 58.2% (93/160) remained in the range of minor increase (between 1 and 6) or minor decrease (between 1 and 7), and 5.6% (9/160) experienced no change. The maximum increase in scores was 17 points (rising from 35 in presemester survey to 52 in post) and the maximum decrease in scores was 30 points (falling from 58 in the presemester survey to 28 in post). These observations indicated that although there were changes in loneliness scores among the majority of participants (154/160), these changes were mostly moderate and rarely caused the participants to fall into a different category of loneliness between the pre- and the postsemester surveys. Due to the relatively stable levels of loneliness, predicting change in loneliness levels was more challenging. However, as described in the following sections, using behavioral features in our machine learning pipeline, we were still able to infer change in loneliness with an accuracy above 88%.

**Figure 5:**
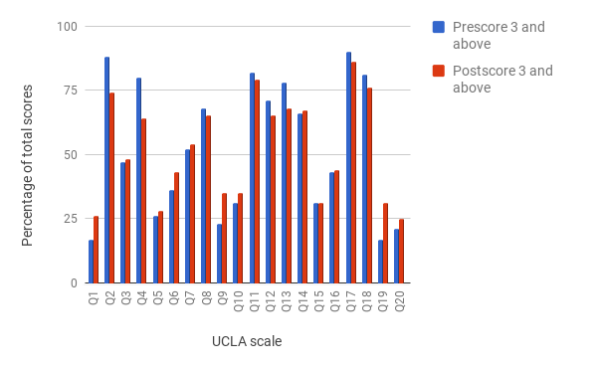
Comparison of pre- and postloneliness ratings of University of California, Los Angeles, questions (Q2, Q4, and Q19 have the largest change in postloneliness ratings). Q: question; UCLA: University of California, Los Angeles.

We also examined the change in scores for each individual question (Table 3). Given our ultimate goal of measuring the power of passive sensing features in distinguishing loneliness behavior, we were curious to know the following: (1) by how much the score of each question changes from presemester to postsemester, (2) what questions had the highest change in score, and (3) whether there were associations between those changes and the behavioral features. Figure 5 shows the percentage of participants rating each question as 3 or above (sometimes or always). For example, the total rating for Q2 (How often do you feel that you lack companionship?) decreased by 14% from presemester to postsemester indicating fewer students felt a lack of companionship at the end of the semester than at the beginning. The largest changes were observed in Q4 (How often do you feel alone?) and QR19 (How often do you feel that there are people you can talk to?) with a total decrease of 16% and an increase of 14%, respectively. Although more analyses are needed to replicate these observations, they may be indicative of changes in specific experiences among students. For example, a decrease in the lack of companionship scores (Q2) may indicate that the participants gained more familiarity with the university environment and were more able to make friends by the end of the semester.

**Table 3:** Statistics of change in loneliness scores measured by University of California, Los Angeles, scale in pre- and postsurveys (N=160).

Change in loneliness score		Participants, n (%)
**Increased score**		
	Overall	75 (47)
	Increase between 1 and 6 points	47 (29)
	Increase more than 6 points	28 (17)
**Decreased score**		
	Overall	76 (47)
	Decrease between 1 and 7 points	46 (29)
	Decrease more than 7 points	30 (19)
Unchanged score		9 (6)

#### Mining Associations Between Overall Loneliness and University of California, Los Angeles, Question Scores

We applied the Apriori algorithm (described in the Methods section) to both the pre- and postsemester survey responses to extract associations between responses to each question and overall loneliness level. Our goal was to identify experiences expressed as responses to each question, which were mostly associated with loneliness in college students, and then examine whether any association between those experiences and passive behavioral features could be observed. We started with a minimum support of 10% and increased it in each iteration to obtain a minimal set of association rules with a maximum support. The optimal minimum support was achieved at 38%, that is, the extracted patterns were observed in at least 38% of the students. We stopped increasing the minimal support after 38% as no rules could be found for a support above that percentage. We kept the minimum confidence at 90%. As shown in Table 4, question 14 (How often do you feel isolated from others?) appears in both pre- and postsemester surveys and indicates that around 42% of students with responses of 3 or 4 to this question also had a high total loneliness score. Responses of 3 or 4 to question 13 (How often do you feel that no one really knows you?) appeared to indicate high total loneliness scores in the presemester survey with 49% support (almost half of the participant population) and 96% confidence. The same association was observed with question 12 (How often do you feel that your relationships with others are not meaningful?) in the postsemester survey with 41% support and 94% confidence.

**Table 4:** Association rules extracted from pre- and postsurvey responses.

Question response level (*sometimes or always),* scale from 1 to 4		Loneliness level (low or high)	Support, % (minimum support = 38%)	Confidence, % (minimum confidence 90%)
**Presurvey**				
	Feeling no one really knows you well (UCLA^a^13 ≥ 3)	High	49	96
	Feeling isolated (UCLA14 ≥ 3)	High	41	95
**Postsurvey**				
	Relationships are not meaningful (UCLA12 ≥ 3)	High	41	94
	Feeling isolated (UCLA14 ≥ 3)	High	42	94

^a^UCLA: University of California, Los Angeles.

#### Detection of Loneliness Level and Change in Loneliness

**Figure 6:**
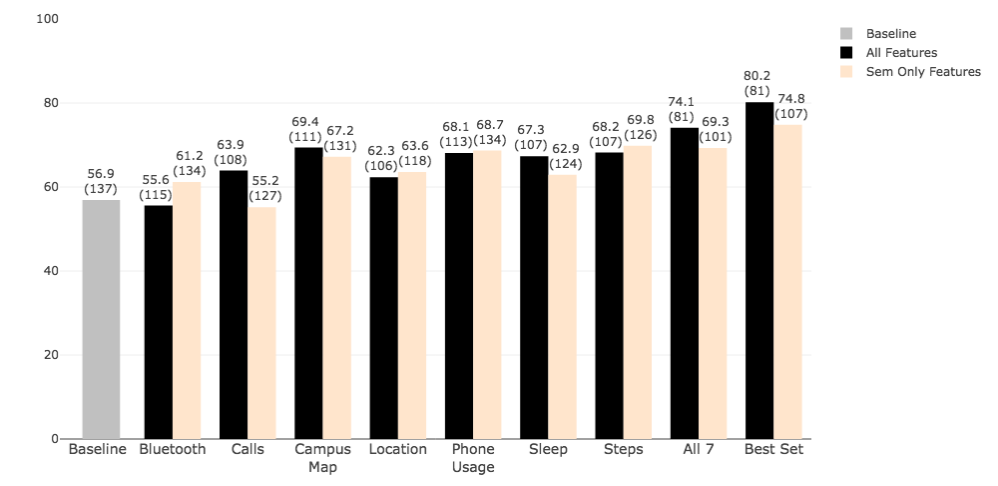
Detection of postloneliness level (high loneliness or low loneliness) using all-epochs features and semester-level features. Each bar shows the accuracy followed by the number of samples used in the analysis in parentheses; the gray bar represents the baseline accuracy as measured by the percentage of samples belonging to the majority class here, that is, high loneliness. Sem: semester.

**Figure 7:**
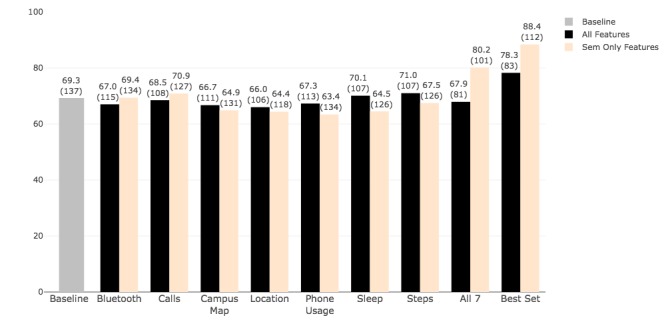
Detection of change in postloneliness level (decreased loneliness, increased loneliness, and unchanged loneliness) using all-epochs features and semester-level features. Each bar shows the accuracy followed by the number of samples used in the analysis in parentheses and baseline accuracy is the percentage of samples belonging to the majority class here, that is, unchanged loneliness. Sem: semester.

We ran our machine learning pipeline to infer 2 outcomes: postloneliness level (low or high) and change in loneliness level (IL, DL, and UL). For both outcomes, we used the set of all-epochs features extracted from all time slices and time slices as described in the processing section, as well as semester-aggregated (semester-level) features. Our goal was to identify a minimal set of features capable of accurately inferring loneliness level. Whereas the all-epochs features provided the opportunity to analyze behavior on a more fine-grained level, the semester-level features provided a reduced set that described the overall behavior of each participant during the semester. Figures 6 and 7 show the accuracy results for both outcomes and their comparison with the baseline (56.9%—the percentage of participants assessed at the HL level in the postsemester survey). The graphs show the accuracy obtained from sensor-specific features (1-feature set), all feature sets combined, and the set that provides the best overall accuracy. For detection of postloneliness, our machine learning pipeline achieved the highest accuracy of 80.2%, using all-epochs features in the best feature set that included call logs, location, location map, screen, sleep, and steps. This accuracy was 6.1% higher than the accuracy obtained from using all 7 feature sets (74.1%) and indicated that including Bluetooth features contributed to performance reduction. The all-epochs-Bluetooth-only features provided 55.6% accuracy, confirming their low prediction power in detecting postloneliness level. Except for Bluetooth, all other feature sets and their combinations achieved a higher (by at least 5.4%) accuracy than the baseline measure. The semester-level features, including the combination of Bluetooth, location, screen, and steps, provided the best set accuracy of 74.8%, which was 5.5% higher than the all features set (69.3%). The semester-level feature set for calls had the lowest accuracy of 55.2% (1.7% lower than baseline). One possible reason for this could be the large number of missing feature values for calls, meaning calls were being made to a large number of individuals that were not on the frequent contacts lists that the participants provided before the study semester. In general, the analysis with all-epochs features included provided better results than the analysis with semester-level features for detecting loneliness level (5.4% higher accuracy). As a point of comparison, we also used the features selected through LASSO in our pipeline to compare the performance (ie, accuracy). The average accuracy obtained from all feature sets was 56.7% which is below the baseline of 56.9%. This indicates that our more sophisticated feature selection approach was effective.

Table 5 summarizes the best results for both inferences distinguishing the performance of the classifiers to infer each class. The recall values (the percentage of correctly classified instances) indicate that the classifier correctly labeled HL instances 76.9% of the time using all-epochs features and 74.6% of the time using semester-level features.

**Table 5:** The performance of models obtained from all-epochs features and semester-level features to detect loneliness level and change in loneliness.

Measure	Loneliness level						Change in loneliness							
	All-epochs features, baseline = 58.5%			Semester-level features, baseline = 58.5%			All-epochs features, baseline = 69.3%				Semester-level features, baseline = 69.3%			
	Average	HL^a^	LL^b^	Average	HL	LL	Average	DL^c^	IL^d^	UL^e^	Average	DL	IL	UL
Accuracy, %	80.2	—^f^	—	74.8	—	—	79.2	—	—	—	88.4	—	—	—
Precision, %	80.3	81.1	79.5	74.6	78.6	70.6	70.2	53.3	71.4	85.7	90.0	90.9	91.6	87.6
Recall, %	80.1	76.9	83.3	74.8	74.6	75.0	67.7	40	71.4	91.6	82.6	75.8	73.3	98.7
F1^g^, %	80.1	78.9	81.3	74.6	76.5	72.7	68.5	45.7	71.4	88.5	81.0	68.9	81.5	92.8
MCC^h^	0.6	—	—	0.5	—	—	0.6	—	—	—	0.7	—	—	—

^a^HL: high loneliness.

^b^LL: low loneliness.

^c^DL: decreased loneliness.

^d^IL: increased loneliness.

^e^UL: unchanged loneliness.

^f^Data not applicable.

^g^F1: harmonic mean of precision and recall.

^h^MCC: a measure of quality of binary classification.

Detection of change in loneliness levels provided slightly different results. Using all-epochs features, the best feature set including calls and screen state achieved 78.3% accuracy, whereas the best set obtained with semester-level features—which included Bluetooth, calls, location, and location map—achieved 88.4% accuracy. In contrast to the postloneliness detection model, where the analysis with all-epochs features provided better results, in these models for detecting change, the semester-level features contributed to higher accuracy using the best set (88.4% with semester only vs 78.3% with all-epochs features) and all 7 sets (80.2% with semester only vs 67.9% with all-epochs features).

For increased loneliness, these recall values were 71.4% and 73.3%, respectively. Although more analyses are needed to replicate these results, we find that even though the all-epochs features provide slightly higher accuracy, these features only gave 2.3% better recall for detecting HL. We also find no statistically significant difference between the accuracies obtained from all-epochs features and semester-only features (*P*=.58). However, the selected all-epochs features during the training and validation process provided a fine-grained set of behavioral patterns associated with the loneliness level that were observed on a week-by-week basis as described below (Tables 6 and 7). These patterns could not be extracted using semester-level features.

The most frequently selected features indicate their high impact in detecting loneliness (see Multimedia Appendix 1 for the list of selected semester-only features in each feature set that appear in more than half of the folds during the cross-validation). However, their selection as part of the analysis pipeline did not answer the question of how these features and their combinations related to loneliness. We, therefore, ran the Apriori algorithm (minimum support=10% and minimum confidence=90%) on these selected features to extract different combinations of behavioral features that were indicative of loneliness. Table 6 summarizes the extracted patterns using the selected features in each analysis (postloneliness detection and change in loneliness).

As shown in Table 6, low frequency of phone usage in certain hours during the weekend and morning hours, as well as spending less time outside of campus and at social-event houses in the evening and night were associated with HL. Recall that support is the percentage of the observed behavior patterns (left column in Tables 6 and 7) in the entire dataset (here the participant population), whereas confidence is the percentage of the samples with that observed pattern that satisfy a certain condition, for example, change in loneliness. For example, the pattern min length of phone usage [weekday] = low and min steps in active bouts [night, weekend] = low and min length of sedentary bouts [night, weekend] = low (third row in Table 6) is observed in 31% of the participants (support) out of which 92% (confidence) experienced a decrease in loneliness at the end of the semester.

As the question 14 on the UCLA scale (feeling of isolation) was most associated with one’s total loneliness score (based on our analysis in previous sections), we also extracted patterns of daily behavior that were associated with scores on that question (feelings of isolation) using the same set of selected features. Table 7 shows the extracted patterns associated with feeling of isolation. For example, the participants with low feelings of isolation spend less time studying in the afternoon on weekends and spend a moderate amount of time in green areas in the morning. Also, higher overall level of activity and steps during the day and evening hours is associated with lower feelings of isolation.

**Table 6:** Extracted patterns showing how combinations of behavioral features selected by the machine learning algorithm are associated with high loneliness and decreased loneliness.

Pattern (features categorized into low, moderate, and high)	Postloneliness
Frequency of first screen unlock between 1 and 2 pm [weekend] = low and frequency of last screen lock between 10 and 11 am [morning] = low and time spent off campus [evening] [weekend] = low and max length of time spent at social-event houses [evening][night] [weekday] = low	Postloneliness = high loneliness Support = 17% Confidence = 92%
Number of scans of the least frequent Bluetooth device belonging to self or others [weekend, week 2017-03-08] = low and number of scans of the least frequent Bluetooth device belonging to others [morning, weekend, week 2017-05-03] = high and last screen lock between 10 and 11 am [morning, weekday, week 2017-04-19] = low and Last screen lock between 2 and 3 pm [afternoon, weekday, week 2017-02-01] [week 2017-03-15] [weekday, week 2017-03-15] = low and time at local cluster 3 [afternoon] [weekend, week 2017-03-08] = low and last screen on between 3 and 4 pm [week 2017-02-01] = low and first screen on between 3 and 4 am [night] [weekday, half semester 2017-03-01] = low	Postloneliness = high loneliness Support = 30% Confidence = 90%
Min length of phone usage [weekday] = low and min steps in active bouts [night, weekend] = low and min length of sedentary bouts [night, weekend] = low	Change = decreased loneliness Support = 31% Confidence = 92%
Last screen unlock between 2 and 3 pm [afternoon, week 2017-03-29] = low and first screen on between 5 and 6 am [weekday, week 2017-03-15] = low and last screen unlock between 1 and 2 am [half semester 2017-03-01] = low and min length of sedentary bouts [morning, weekday] = low and first screen unlock between 5 and 6 pm [week 2017-02-08] = low and minimum length of sleep duration [weekend, half semester 2017-03-01] = low	Change = decreased loneliness Support = 50% Confidence = 90%

**Table 7:** Extracted behavioral patterns associated with the feeling of isolation.

Pattern (features categorized into low, medium, and high)	UCLA^a^14 level	Support, % (minimum support = 10%)	Confidence, % (minimum confidence = 90%)
Number of scans of the least frequent Bluetooth device belonging to others [morning, weekend] = low and study duration [afternoon, weekend] = low and minimum stay in green areas [morning] = moderate	Feeling of isolation = low	18	92
Total sleep [morning, week 2017-03-01] = moderate and number of scans of the least frequent Bluetooth device belonging to others [evening, weekend, week 2017-02-01] [weekend, week 2017-02-22] [afternoon, weekday, week 2017-01-25] = moderate and number of scans of the least frequent Bluetooth device [night, weekday, half semester 2017-03-01] [night, week 2017-01-18] [night, weekday, half semester 2017-01-18] = low and first screen unlock between 10 and 11 am [weekday, week 2017-04-26] = low	Feeling of isolation = low	30	90
Time at frequent locations [afternoon, weekday] = low	Change = low to high	28	92
First screen unlock between 3 and 4 am [night, weekend] = low and average steps in active bouts [morning, weekend] = high	Change = high to low	28	92
Minimum length of sleep [weekend, week 2017-01-18] = moderate and number of scans of the least frequent Bluetooth device [night, weekday, half semester 2017-03-01] [night, weekday, half semester 2017-01-18] = low and first screen on between noon and 1 pm [afternoon, weekend, week 2017-04-26] = low and first screen on between 7 and 8 am [weekday, week 2017-03-15] = low and last screen lock between midnight and 1 am [half semester 2017-03-01] = low and first screen on between 3 and 4 am [night, weekday, week 2017-03-08] = low	Change = high to low	33	93

^a^UCLA: University of California, Los Angeles.

## Discussion

### Principal Findings and Comparison With Previous Research

This study reported a 3-fold analysis of loneliness among college students, exploring the potential of passively collected sensing data from mobile and wearable devices to estimate loneliness and identify behavioral data features associated with loneliness. Results showed that fine-grained behavioral features extracted from mobile and wearable time series data can detect low and high levels of loneliness with high accuracy and that these features can distinguish the behavior of students with high levels of loneliness from those with low levels of loneliness. For example, results showed that students with high levels of loneliness spend less time off campus and socialize less in the evening during weekends than students with low levels of loneliness.

We extend existing research on the study of loneliness in 5 ways. First, we collected behavioral data from a substantially large sample (n=160) of college students for a period of 16 weeks, a longer period of time compared with the current state of the research [11,12,14]. This provided the opportunity to analyze long-term behavior associated with loneliness through pattern mining and observe changes in behavior that are associated with changes in loneliness. Second, we extracted a much larger set of behavioral features from raw data collected on smartphones and wearable devices (77,805 features) and showed their impact in detecting the level of loneliness and the level of change in loneliness detection. The features provided a lens for observing more fine-grained behavior patterns associated with loneliness. Third, for the first time, we presented the associations between the level of loneliness and the combinations of behavioral features. This analysis provided a set of objectively extracted patterns that described behaviors associated with loneliness. Fourth, in addition to the overall level of loneliness, we mined associations between levels of loneliness and responses for each question. We found strong association patterns between pre- and postloneliness scores and the UCLA scale question related to feelings of isolation. We consequently mined associations between responses to this question and behavioral features and found that high level of activity and steps during the day and evening hours were associated with lower feelings of isolation. These results are important as they may provide objective measurements for experiences associated with different dimensions of loneliness (as assessed by specific questions on the UCLA scale) in the form of combined behavioral features. Finally, through a machine learning analysis, we estimated overall levels of loneliness and change in loneliness with a high accuracy of 80.2% and 88.4%, respectively. Other than the study by Pulekar et al [11] that analyzed 2 weeks of data from 9 students using a small set of features from smartphones only and the study by Sanchez et al [12] that inferred different types of loneliness in 12 older adults using one week of mobile data, we are unaware of any existing study to detect loneliness from longitudinal passive sensing data using machine learning.

Our sample of college students had HL scores, consistent with the latest US loneliness index [2], suggesting that this age group experienced the most loneliness of all generations surveyed. We also found that the feeling of isolation was a strong and consistent indicator of loneliness in both pre- and postsemester surveys. The feeling of nobody really knows you was a stronger indicator in the presemester survey. This may be a result of first-year college students still trying to form bonds with their classmates. On the other hand, relationships lacking meaning was associated strongly with the postsemester loneliness scores, which might indicate that students were not having meaningful relationships with their peers. Although mining these associations is a novel approach and the observations are interesting, future analyses on similar datasets must be conducted to confirm these results.

We extracted a rich set of day-level features from the smartphone and Fitbit reflecting activity and mobility, communication, sleep, and phone usage patterns. We also generated a set of aggregated features on the semester-level. We used these sets of features in a machine learning pipeline to infer levels of loneliness and change in loneliness scores. We trained and evaluated an ensemble classifier on a leave-one-student-out cross-validation manner to explore how accurately the level of postsemester loneliness, as well as change in loneliness scores, could be estimated from passive behavioral features. We reported the average results of cross-validation for each outcome. We included feature selection as part of the training process to acquire a set of behavioral features that were repeatedly selected as impactful for the majority of students. Our analysis showed more fine-grained behavioral features were better at identifying the overall level of loneliness (80.2% accuracy), whereas the aggregated semester-level features better distinguished the level of change (88.4% accuracy). Although the higher accuracy achieved with the all-epochs features was modest considering the large number of features used in the pipeline, the analysis provided a set of features that could be used to mine detailed association patterns of loneliness on a week-by-week basis.

Our pattern mining approach, using the selected features, showed that patterns and timings of phone usage combined with spending less time off campus and at social-event houses during evening hours on weekends were most indicative of HL in college students. It also showed that lower phone usage and less activity after midnight was associated with a decrease in loneliness at the end of the semester. In addition to lower phone usage during night hours, we found that a high level of activity (especially in morning hours), less time studying on weekends, and spending more time in green areas were associated with feeling less isolated. These findings are consistent with the results of the study by Wang et al [14] that found negative correlations between loneliness and activity duration for day and evening. However, to our knowledge, this is the first study that reports on combinations of behavioral features observed in a study population.

### Limitations

This study provides insights into understanding loneliness through passive behavioral features. However, it has a number of limitations. First, although we purposely chose university students as our study participants, our results may only generalize to this population. Second, despite the 1-semester duration of this study (which is considerably longer than most existing research on loneliness), studies with longer-term data collection periods may reveal additional patterns in behavior that could not be observed in one semester. Although our analyses provide novel and interesting insights into understanding loneliness behavior through the objective lens of passive sensing, more analyses on the same type of data are needed to provide enough evidence for generalizability of our results. Third, technical issues resulted in a large amount of missing data from many participants that had to be removed from the machine learning analysis, considerably reducing the size of the dataset. Although missing data is a common problem in data analysis, more careful and conservative planning and more stable software may reduce the risk of missing data. Fourth, as we could not find well-known methods in the literature for choosing our thresholds (eg, the cutoff score to indicate the level of loneliness or the number of steps for identifying activity bouts), we made those choices in consultation with the psychologists on our team. We understand that different thresholds may provide different results. However, our goal in this study was not to find the optimal thresholds but rather understand the experience of loneliness in college students and explore the feasibility of using passive sensing to detect loneliness and behavior patterns associated with it. Fifth, although students were instructed to wear the Fitbit on their nondominant hand, because of the nature of our study that collects data in the wild, we did not have much control over the ways students wore the Fitbit nor could we track whether or not the Fitbit was worn or charged. We acknowledge that these factors may affect the measurements related to activities and sleep. However, this is a technical challenge that we face with data collection in the wild and we are working on developing solutions that provide an accurate estimation of user’s activity despite the variations in their wearing patterns. Finally, we developed a machine learning pipeline that could handle a large number of features, select a stable set of features, and use them in the training and validation process. For the first time, we also showed the potential of using data mining to explore the combination of behavioral features that are associated with a health outcome. The developed pipeline and our data mining approach can be adapted by the research community as a generic framework for studies assessing other outcomes. However, we acknowledge that the results obtained through the pipeline and Apriori are highly dependent on the parameter settings and processing steps and results may vary with different data and feature sets.

We plan to advance our machine learning pipeline to test different feature selection and learning algorithms and to automatically find the optimized parameters. Our future plans revolve around addressing some of the above limitations including more systematic threshold setting for both feature extraction and the outcome measure (loneliness level). We plan to experiment with multiple categories and thresholds in future study. We also plan to add more analyses to study the relationship among loneliness, depression, stress, and other mental health outcomes.

### Conclusions

Our findings highlight the feasibility of using ubiquitous smartphone and wearable sensors to passively detect loneliness in college students and identify the behavioral patterns associated with loneliness. The findings suggest an approach for passively sensing loneliness and providing opportunities that could reduce loneliness by, for example, notifying family members and friends to provide social support, connecting the person with similar people, or recommending activities, such as going off campus, spending time in green areas, or going to social events of interest. Building interventions based on empirical findings regarding the experience of loneliness could meaningfully affect students’ well-being.

## Appendix

Multimedia Appendix 1 List of semester-only features that were selected in our model for more than half of the folds during cross-validation.

## Abbreviations

API: app programming interface
DBSCAN: density-based spatial clustering of applications with noise
DL: decreased loneliness
FEC: feature extraction component
GPS: global positioning system
HL: high loneliness
ID: identity document
IL: increased loneliness
LASSO: least absolute shrinkage and selection operator
LL: low loneliness
NRLR: nested randomized logistic regression
SMS: short message service
UCLA: University of California, Los Angeles
UL: unchanged loneliness
