# Supplementary material for: Identifying Behavioral Phenotypes of Loneliness and Social Isolation with Passive Sensing: Statistical Analysis, Data Mining and Machine Learning of Smartphone and Fitbit Data
Source: JMIR Mhealth Uhealth. 2019 Jul 24;7(7):e13209. doi: 10.2196/13209 (PMC6685126; doi:10.2196/13209)
Supplement: Multimedia Appendix 1 [file mhealth_v7i7e13209_app1.pdf]

APPENDIX

Contained in this Appendix are the list of semester-only features that were selected in our model, for more than half of the folds during cross-validation.

Coding schema:

blue = bluetooth
call = calls
loc = location
locMap = location map
screen = screen
slp = sleep
steps = steps
mo = morning
af = afternoon
ev = evening
ni = night
wkdy = weekday
wkend = weekend
place_001 = one of top 2 social-event houses
place_002 = one of top 3 social-event houses
place_003 = social-event houses
place_004 = apartments
place_005 = halls
place_006 = athletic
place_007 = green area
place_008 = academic buildings
Outside = off-campus

Table 7. List of most selected features that appear in more than half of the folds per feature set in the machine learning pipeline using the semester-only

Feature	Num of folds
FG6_f_blue_num_scans_of_least_frequent_device_of_others_mo_wkend	134
FG2_f_blue_number_unique_devices_of_self_wkdy	134
FG3_f_blue_std_num_scans_of_all_devices_of_self_wkend	134
FG6_f_blue_num_scans_of_least_frequent_device_mo_wkend	134
FG15_f_blue_num_scans_of_least_frequent_device_ni_wkend	134
FG10_f_blue_std_num_scans_of_all_devices_of_self_ev	134

FG9_f_blue_std_num_scans_of_all_devices_of_self_af_wkend	134
FG8_f_blue_number_unique_devices_of_self_af_wkdy	134
FG12_f_blue_std_num_scans_of_all_devices_of_others_ev_wkend	134
FG14_f_blue_sum_num_scans_of_all_devices_of_self_ni_wkdy	134
FG6_f_blue_number_unique_devices_of_self_mo_wkend	134
FG14_f_screen_first_on_Hour_4_ni_wkdy	134
FG3_f_screen_first_unlock_Hour_8_wkend	134
FG1_f_screen_last_unlock_Hour_11	134
FG3_f_screen_first_unlock_Hour_13_wkend	134
FG3_f_screen_last_on_Hour_1_wkend	134
FG8_f_blue_num_scans_of_least_frequent_device_af_wkdy	133
FG14_f_blue_number_unique_devices_of_self_ni_wkdy	133
FG9_f_blue_num_scans_of_most_frequent_device_of_self_af_wkend	133
FG14_f_blue_num_scans_of_least_frequent_device_of_others_ni_wkdy	133
FG8_f_blue_num_scans_of_least_frequent_device_of_others_af_wkdy	133
FG14_f_blue_num_scans_of_least_frequent_device_ni_wkdy	133
FG8_f_blue_std_num_scans_of_all_devices_of_self_af_wkdy	133
FG15_f_blue_num_scans_of_least_frequent_device_of_others_ni_wkend	133
FG1_f_screen_last_on_Hour_11	132
FG8_f_screen_last_on_Hour_5_af_wkdy	132
FG7_f_blue_number_unique_devices_of_self_af	131
FG14_f_blue_num_scans_of_least_frequent_device_of_self_ni_wkdy	131
FG5_f_blue_std_num_scans_of_all_devices_of_self_mo_wkdy	131

FG7_f_locMap_study_duration_minutes_af	131
FG12_f_locMap_minutes_at_place_outside_ev_wkend	131
FG9_f_locMap_study_duration_minutes_af_wkend	131
FG12_f_locMap_mean_bout_at_place_007_ev_wkend	131
FG12_f_locMap_study_duration_minutes_ev_wkend	131
FG12_f_locMap_min_bout_at_place_007_ev_wkend	131
FG11_f_locMap_mean_bout_at_place_001_ev_wkdy	131
FG14_f_locMap_mean_bout_at_place_001_ni_wkdy	131
FG12_f_locMap_num_bouts_30min_or_more_at_place_outside_ev_wkend	131
FG13_f_blue_num_scans_of_least_frequent_device_of_self_ni	130
FG11_f_blue_num_scans_of_most_frequent_device_of_self_ev_wkdy	130
FG11_f_locMap_max_bout_at_place_001_ev_wkdy	130
FG3_f_locMap_num_bouts_20min_or_more_at_place_002_wkend	130
FG7_f_locMap_min_bout_at_place_007_af	130
FG15_f_screen_last_unlock_Hour_0_ni_wkend	130
FG4_f_screen_last_lock_Hour_4_mo	130
FG15_f_call_number_incoming_calls_ni_wkend	127
FG11_f_steps_max_steps_ev_wkdy	126
FG13_f_steps_max_length_sedentary_bout_minutes_ni	126
FG8_f_steps_max_steps_af_wkdy	126
FG10_f_steps_max_step_active_bout_ev	126
FG3_f_steps_max_length_sedentary_bout_minutes_wkend	126
FG10_f_steps_max_steps_ev	126

FG6_f_steps_avg_length_sedentary_bout_minutes_mo_wkend	126
FG14_f_steps_max_length_sedentary_bout_minutes_ni_wkdy	126
FG12_f_steps_max_length_sedentary_bout_minutes_ev_wkend	126
FG5_f_steps_avg_step_active_bout_mo_wkdy	126
FG5_f_steps_avg_length_active_bout_minutes_mo_wkdy	126
FG13_f_steps_max_step_active_bout_ni	126
FG12_f_steps_avg_length_active_bout_minutes_ev_wkend	126
FG10_f_steps_avg_length_active_bout_minutes_ev	126
FG13_f_steps_max_steps_ni	126
FG9_f_steps_avg_length_sedentary_bout_minutes_af_wkend	126
FG11_f_steps_avg_length_active_bout_minutes_ev_wkdy	126
FG6_f_steps_max_length_active_bout_minutes_mo_wkend	126
FG3_f_steps_avg_length_sedentary_bout_minutes_wkend	126
FG15_f_steps_max_length_sedentary_bout_minutes_ni_wkend	126
FG9_f_steps_sum_steps_af_wkend	126
FG12_f_steps_max_steps_ev_wkend	126
FG15_f_steps_avg_length_sedentary_bout_minutes_ni_wkend	125
FG12_f_steps_max_length_active_bout_minutes_ev_wkend	125
FG8_f_steps_max_length_sedentary_bout_minutes_af_wkdy	125
FG2_f_steps_max_length_active_bout_minutes_wkdy	125
FG13_f_steps_max_length_active_bout_minutes_ni	125
FG14_f_blue_number_unique_devices_of_others_ni_wkdy	124
FG14_f_locMap_max_bout_at_place_001_ni_wkdy	124

FG13_f_slp_avg_length_bout_awake_ni	124
FG4_f_slp_start_time_max_bout_restless_mo	124
FG6_f_slp_start_time_min_bout_restless_mo_wkend	124
FG10_f_slp_start_time_max_bout_asleep_ev	124
FG2_f_slp_end_time_min_bout_totalsleep_wkdy	124
FG3_f_slp_end_time_max_bout_awake_wkend	124
FG6_f_slp_end_time_min_bout_totalsleep_mo_wkend	124
FG3_f_slp_end_time_max_bout_totalsleep_wkend	124
FG11_f_slp_start_time_min_bout_restless_ev_wkdy	124
FG15_f_slp_end_time_min_bout_totalsleep_ni_wkend	124
FG4_f_slp_num_totalsleep_bouts_mo	124
FG3_f_slp_end_time_min_bout_totalsleep_wkend	124
FG4_f_slp_start_time_max_bout_totalsleep_mo	124
FG6_f_slp_min_length_bout_asleep_mo_wkend	124
FG12_f_slp_sum_length_bout_awake_ev_wkend	124
FG6_f_slp_start_time_max_bout_restless_mo_wkend	124
FG14_f_slp_start_time_min_bout_totalsleep_ni_wkdy	124
FG2_f_slp_sleep_eff_general_weak_wkdy	124
FG15_f_slp_max_length_bout_awake_ni_wkend	124
FG13_f_slp_end_time_max_bout_restless_ni	124
FG14_f_slp_start_time_max_bout_totalsleep_ni_wkdy	124
FG3_f_slp_end_time_min_bout_asleep_wkend	124
FG5_f_slp_min_length_bout_awake_mo_wkdy	124

FG10_f_slp_max_length_bout_asleep_ev	124
FG10_f_slp_start_time_min_bout_restless_ev	124
FG15_f_slp_end_time_max_bout_awake_ni_wkend	124
FG3_f_slp_start_time_max_bout_totalsleep_wkend	124
FG6_f_slp_start_time_min_bout_asleep_mo_wkend	124
FG1_f_slp_end_time_max_bout_totalsleep	124
FG1_f_slp_end_time_max_bout_asleep	124
FG13_f_slp_start_time_max_bout_totalsleep_ni	124
FG11_f_slp_end_time_max_bout_totalsleep_ev_wkdy	124
FG15_f_slp_avg_length_bout_awake_ni_wkend	124
FG2_f_slp_max_length_bout_asleep_wkdy	124
FG3_f_slp_start_time_min_bout_asleep_wkend	124
FG13_f_slp_end_time_max_bout_awake_ni	124
FG7_f_slp_min_length_bout_asleep_af	124
FG3_f_slp_min_length_bout_totalsleep_wkend	124
FG1_f_steps_avg_length_sedentary_bout_minutes	124
FG12_f_blue_number_unique_devices_ev_wkend	123
FG12_f_locMap_max_bout_at_place_007_ev_wkend	123
FG1_f_screen_first_unlock_Hour_7	123
FG5_f_slp_num_restless_bouts_mo_wkdy	123
FG6_f_slp_start_time_max_bout_totalsleep_mo_wkend	123
FG4_f_slp_start_time_min_bout_totalsleep_mo	123
FG2_f_slp_start_time_max_bout_asleep_wkdy	123

FG7_f_slp_start_time_max_bout_asleep_af	123
FG11_f_slp_avg_length_bout_restless_ev_wkdy	123
FG12_f_slp_num_awake_ev_wkend	123
FG4_f_steps_avg_length_active_bout_minutes_mo	123
FG4_f_slp_end_time_min_bout_totalsleep_mo	122
FG5_f_slp_end_time_max_bout_restless_mo_wkdy	122
FG11_f_slp_min_length_bout_restless_ev_wkdy	122
FG5_f_slp_avg_length_bout_awake_mo_wkdy	122
FG5_f_screen_last_lock_Hour_4_mo_wkdy	121
FG2_f_steps_max_length_sedentary_bout_minutes_wkdy	121
FG4_f_steps_max_steps_mo	121
FG1_f_slp_num_totalsleep_bouts	120
FG13_f_slp_max_length_bout_awake_ni	120
FG3_f_slp_end_time_min_bout_awake_wkend	120
FG3_f_slp_sum_length_bout_awake_wkend	120
FG2_f_slp_end_time_max_bout_totalsleep_wkdy	120
FG6_f_blue_number_unique_devices_of_others_mo_wkend	119
FG5_f_slp_start_time_min_bout_totalsleep_mo_wkdy	119
FG5_f_slp_start_time_min_bout_restless_mo_wkdy	119
FG12_f_loc_home_stay_time_percent_10m_ev_wkend	118
FG11_f_slp_avg_length_bout_awake_ev_wkdy	118
FG5_f_slp_avg_length_bout_asleep_mo_wkdy	118
FG14_f_slp_min_length_bout_awake_ni_wkdy	118

FG13_f_slp_start_time_min_bout_totalsleep_ni	117
FG3_f_loc_mean_len_stay_at_clusters_in_minutes_wkend	116
FG3_f_loc_std_len_stay_at_clusters_in_minutes_wkend	116
FG12_f_slp_num_0_ev_wkend	116
FG6_f_slp_start_time_max_bout_asleep_mo_wkend	115
FG12_f_slp_min_length_bout_asleep_ev_wkend	114
FG3_f_slp_num_awake_wkend	114
FG13_f_slp_min_length_bout_awake_ni	114
FG5_f_steps_max_length_active_bout_minutes_mo_wkdy	114
FG5_f_slp_start_time_max_bout_totalsleep_mo_wkdy	113
FG5_f_slp_num_asleep_bouts_mo_wkdy	113
FG2_f_screen_last_on_Hour_22_wkdy	110
FG15_f_slp_min_length_bout_totalsleep_ni_wkend	110
FG15_f_steps_max_steps_ni_wkend	110
FG10_f_locMap_study_duration_minutes_ev	109
FG4_f_slp_min_length_bout_asleep_mo	109
FG12_f_steps_max_step_active_bout_ev_wkend	109
FG9_f_steps_avg_step_active_bout_af_wkend	109
FG5_f_slp_min_length_bout_asleep_mo_wkdy	108
FG3_f_slp_start_time_min_bout_awake_wkend	108
FG3_f_slp_min_length_bout_awake_wkend	107
FG4_f_steps_num_active_bout_mo	106
FG12_f_steps_num_active_bout_ev_wkend	106

FG12_f_steps_num_sedentary_bout_ev_wkend	105
FG3_f_slp_end_time_max_bout_asleep_wkend	104
FG11_f_steps_max_length_sedentary_bout_minutes_ev_wkdy	104
FG7_f_steps_max_steps_af	104
FG3_f_steps_max_steps_wkend	104
FG1_f_blue_avg_num_scans_of_all_devices_of_others	103
FG3_f_screen_first_on_Hour_8_wkend	102
FG10_f_steps_max_length_sedentary_bout_minutes_ev	98
FG5_f_steps_max_length_sedentary_bout_minutes_mo_wkdy	98
FG3_f_slp_num_totalsleep_bouts_wkend	96
FG14_f_slp_num_awake_ni_wkdy	95
FG4_f_locMap_min_bout_at_place_007_mo	93
FG12_f_slp_max_length_bout_awake_ev_wkend	91
FG5_f_slp_sleep_eff_general_weak_mo_wkdy	90
FG7_f_steps_max_length_active_bout_minutes_af	90
FG15_f_blue_number_unique_devices_of_others_ni_wkend	89
FG5_f_locMap_std_bout_at_place_006_mo_wkdy	89
FG8_f_locMap_mean_bout_at_place_outside_af_wkdy	88
FG2_f_slp_num_totalsleep_bouts_wkdy	88
FG6_f_slp_end_time_min_bout_asleep_mo_wkend	87
FG14_f_slp_sum_length_bout_awake_ni_wkdy	86
FG7_f_slp_num_restless_af	85
FG7_f_slp_sum_length_bout_restless_af	85

FG12_f_steps_sum_steps_ev_wkend	85
FG11_f_slp_max_length_bout_awake_ev_wkdy	83
FG9_f_steps_max_length_sedentary_bout_minutes_af_wkend	83
FG3_f_slp_max_length_bout_awake_wkend	82
FG1_f_slp_start_time_min_bout_totalsleep	80
FG11_f_steps_sum_steps_ev_wkdy	79
FG11_f_slp_start_time_max_bout_asleep_ev_wkdy	78
FG11_f_locMap_min_bout_at_place_001_ev_wkdy	77
FG12_f_blue_number_unique_devices_of_others_ev_wkend	76
FG14_f_blue_avg_num_scans_of_all_devices_of_others_ni_wkdy	73
FG5_f_slp_num_awake_bouts_mo_wkdy	73
FG11_f_slp_end_time_min_bout_totalsleep_ev_wkdy	71
FG8_f_steps_sum_steps_af_wkdy	71
FG2_f_slp_num_awake_bouts_wkdy	67
FG6_f_steps_max_steps_mo_wkend	67
FG3_f_locMap_num_bouts_30min_or_more_at_place_002_wkend	66
FG14_f_slp_end_time_max_bout_asleep_ni_wkdy	66
FG15_f_slp_end_time_min_bout_awake_ni_wkend	62
